# Comparison of Nasal Epithelial Smoking-Induced Gene Expression on Affymetrix Exon 1.0 and Gene 1.0 ST Arrays

**DOI:** 10.1155/2013/951416

**Published:** 2013-02-17

**Authors:** Xiaoling Zhang, Marc E. Lenburg, Avrum Spira

**Affiliations:** ^1^Division of Computational Biomedicine, Boston University School of Medicine, 72 East Concord Street, E631, Boston, MA 02118, USA; ^2^Division of Intramural Research, National Heart, Lung and Blood Institute, The NHLBI's Framingham Heart Study, 73 Mt. Wayte Avenue Suite 2, Framingham, MA 01702, USA; ^3^Pulmonary Center, Boston University Medical Center, 715 Albany Street, Boston, MA 02118, USA

## Abstract

We have previously defined the impact of tobacco smoking on nasal epithelium gene expression using Affymetrix Exon 1.0 ST arrays. In this paper, we compared the performance of the Affymetrix GeneChip Human Gene 1.0 ST array with the Human Exon 1.0 ST array for detecting nasal smoking-related gene expression changes. RNA collected from the nasal epithelium of five current smokers and five never smokers was hybridized to both arrays. While the intersample correlation within each array platform was relatively higher in the Gene array than that in the Exon array, the majority of the genes most changed by smoking were tightly correlated between platforms. Although neither array dataset was powered to detect differentially expressed genes (DEGs) at a false discovery rate (FDR) <0.05, we identified more DEGs than expected by chance using the Gene ST array. These findings suggest that while both platforms show a high degree of correlation for detecting smoking-induced differential gene expression changes, the Gene ST array may be a more cost-effective platform in a clinical setting for gene-level genomewide expression profiling and an effective tool for exploring the host response to cigarette smoking and other inhaled toxins.

## 1. Introduction

Cigarette smoking is well recognized as the major cause of lung cancer and chronic obstructive pulmonary disease (COPD) [1]; however, only 10%–20% of smokers actually develop these diseases [2]. Further, it is unclear why some smokers remain healthy while others are still at high risk decades even after they have quit [3]. Unfortunately, there are currently no effective tools for identifying current and former smokers at highest risk for developing tobacco-related lung diseases. 

Based on the concept that cigarette smoking creates a “field of injury” in epithelial cells throughout the respiratory tract, we have previously measured smoking-induced gene expression changes in bronchial airway epithelial cells obtained via bronchoscopy among healthy never, former, and current smokers using Affymetrix HG-U133A Array [4, 5]. Further, we developed a profile of bronchial airway gene expression that can distinguish smokers with and without lung cancer and could serve as an early diagnostic biomarker for disease [6]. However, the invasiveness of bronchoscopy prevents it from being used as a screening tool for assessing smoking-induced lung cancer risk in large population studies. Most recently, utilizing Affymetrix Human Exon 1.0 ST (sense target) Array, we have demonstrated that smoking induces mostly similar gene-expression changes in both nasal and bronchial epithelium [7], suggesting that nasal epithelium could be a relatively noninvasive surrogate to measure physiological responses to cigarette smoking in several scenarios, for example, to estimate lung cancer risk of smokers in large-scale epidemiological studies, to detect second-hand smoking effects among children and adults, and to examine short- and long-term smoking damage in smoking cessation studies which require repeatedly collecting multiple samples from the same individuals. 

In the previously mentioned comparison study, the Human Exon 1.0 ST array, the first in the whole-transcripts- (WTs-) based array family, was applied. This array contains >5 million 25 mer probes, interrogating 1 million known and predicted exons [8], resulting in a comprehensive gene-level analysis, alternative splicing analysis, and novel exon and transcript detection. However, this platform is cost prohibitive, and the majority of expression biomarker applications only focus on known and manually curated genes. The Human Gene 1.0 ST array (Affymetrix, Santa Clara, CA) uses a subset of the same probes on the Human Exon 1.0 ST array to interrogate the more focused, well-annotated content at the gene level. Probes are also designed across the entire length of the genomic locus to provide a robust and accurate representation of total transcription activity for genes from RefSeq, Ensembl, and putative complete CDS GenBank transcripts. Predicted and discovery-oriented content from the Human Exon 1.0 ST array has been dropped; together this permits the use of a smaller, more affordable chip format for the Human Gene 1.0 ST array. The end result is a single focused gene-level expression array interrogating 28,869 well-annotated full-length genes with 764,885 distinct probes (on average, 28 probes per gene). Although the Gene ST array uses sparser probe coverage than the Exon array, it provides the same advantages of the Exon array for gene-level analysis and for partially degraded mRNA since both are WT-based arrays *(probes are distributed along the whole length of the gene).* Furthermore, the Gene ST array offers several advantages over the Exon array: (1) lower cost, easier to analyze due to having more than 6.5 times less, and more focused content (764,885 probes compared to 5 million on Exon arrays); (2) requiring 10 times less starting RNA materials (100 ng of total RNAs compared to 1 *μ*g for Exon arrays), which will be tremendously beneficial for clinical studies where limited amounts of RNAs are available. 

There are very limited data comparing the robustness and reproducibility of the Gene ST and Exon arrays, particularly in the setting of clinical samples. Previously, we have shown that there was a high correspondence of smoking-induced gene expression changes between the Human Exon and U133 arrays by hybridizing the same bronchial epithelial samples to both platforms [8]. Other studies have demonstrated a reasonable correlation in signals for genes that were differentially expressed between tissue types (heart versus brain) by assaying those tissue samples in parallel on both Exon and Gene ST arrays [9]. In this study, in order to estimate the performance of these two platforms on detecting differentially expressed genes in our nasal epithelial cells, we first performed a systematic comparison of the gene signal estimations from the Human Exon and Gene ST arrays by hybridizing the same nasal epithelial RNA samples obtained from smokers and nonsmokers to both arrays. Then, utilizing different chip description files (CDFs) for the preprocessing, we evaluated the impact of preprocessing and probe selection on the performance of these two array systems. Our data suggest a high degree of correlation between both platforms for detecting smoking-induced differential gene expression changes, with the Gene ST array being a more cost-effective and flexible platform in a clinical setting for the genomewide study of gene-level expression profiling.

## 2. Material and Methods

### 2.1. Study Population

We recruited 5 healthy never smokers and 5 current smokers for the study at Boston Medical Center. Nonsmokers with a history of significant second-hand environmental cigarette exposure, individuals with respiratory symptoms, or regular use of inhaled medications was excluded. For each participant, a detailed smoking history was obtained including, for smokers, cumulative tobacco exposure (measured in pack-years), age when they began smoking, and for all participants the extent of second-hand tobacco exposure. All individuals were screened with routine chest X-ray and spirometry and were excluded if there was evidence of pulmonary pathology. The study was approved by the Institutional Review Board of Boston Medical Center, and all participants provided written informed consent. Of note, there were no significant differences (*P* > 0.05) in age, race, and gender between 5 nonsmokers and 5 smokers in this study.

### 2.2. Sample Collection

Nasal epithelial cells were collected by brushing the inferior turbinate of the nose as previously described [7]. Briefly, the right nare was lavaged with 1 cc of 1% lidocaine. A nasal speculum (Bionix, Toledo, OH) then spread the nare while a standard cytology brush was inserted underneath the inferior nasal turbinate. The brush was rotated in place for 3 seconds, removed, and immediately placed in 1 mL RNAlater (Qiagen, Valencia, CA). After storage at 4°C, RNA was isolated via Qiagen RNeasy Mini Kits as per the manufacturer protocol. Integrity of the RNA samples was assessed by Agilent BioAnalyzer, and purity of the RNA was confirmed using a NanoDrop spectrophotometer. 

4-5 mL of blood was obtained from the study participants for determination of plasma cotinine. Samples were centrifuged, and 2.2 mL of plasma was stored at −80°C, and then shipped on dry ice to the San Francisco Division of Clinical Pharmacology and Experimental Therapeutics, University of California. Gas chromatography (quantitation limit = 10 ng/mL) or liquid chromatography-tandem mass spectrometry (quantitation limit = .02 ng/mL) was used to analyze samples for the presence of this nicotine metabolite from self-reported current and never smokers, respectively.

### 2.3. Microarray Data Acquisition and Preprocessing

#### 2.3.1. Affymetrix Human Exon 1.0 ST Array

1 *μ*g of total RNA from the nasal epithelium samples was used as the starting material. Ribosomal RNA was first removed using the RiboMinus Human/Mouse Transcriptome Isolation Kit (Invitrogen, Carlsbad, CA). This treated RNA was then converted to cDNA and subsequently processed, labeled, and hybridized onto the Exon arrays as previously described [7]. Following hybridization, each array was washed and stained according to the standard Affymetrix protocol. The stained array was scanned using an Affymetrix GeneChip Scanner 3000, resulting in a raw data CEL file for each array. The approximately 17,800 empirically supported transcripts (RefSeq and full-length GenBank mRNAs) were used for gene-level analysis. About 230,000 “core” exon-level probesets on the Exon array were mapped to these core transcripts with a high degree of confidence. Gene-level expression values were derived from CEL files by quantile sketch normalization using the model-based Robust Multichip Average (RMA) method [10] as implemented in the Exon Array Computational Tool (ExACT) software package (Affymetrix, Santa Clara, CA). The gene annotations used for each probe set were from the annotation file obtained from Affymetrix (http://www.affymetrix.com/). 

#### 2.3.2. Affymetrix Human Gene 1.0 ST Array

Between 100 and 300 *n*g of total RNA was processed, labeled, and hybridized to Gene 1.0 ST arrays. The protocol for the Gene ST array was essentially the same as that for the Exon array, so they were combined in one protocol. The RMA method in the ExACT package was used for background adjustment, normalization, and probe-level summarization of the microarray samples. Although the Gene ST array contains 28,869 genes with support from RefSeq, Ensembl, and putative complete CDS GenBank, to be consistent with the previous analysis for the Exon array, we used the CDF provided by Affymetrix (the default parameter in ExACT) to preprocess the raw data. This led to 19,734 gene-level probe sets with putative full-length transcript support in the GenBank and RefSeq databases (http://www.affymetrix.com/estore/browse/products.jsp;jsessionid=7F717F80BE253ABBF6155B16AC95C6F9?navMode=34000&productId=131453&navAction=jump&aId=productsNav#1_1).

### 2.4. Microarray Data Analysis

All of these 10 samples were processed in one batch for each array type. After preprocessing, we applied the principal components analysis (PCA) and the relative log expression (RLE) signal to check the quality of the arrays. Not surprisingly, all of the samples passed the quality metrics. For comparing the fold change of smoking-induced gene expression changes on both arrays, we considered the RefSeq mappings as a nonredundant and relatively complete database of transcripts. In total, 17,881 core transcripts with annotation on the Exon array were mapped to 19,734 transcripts with annotation on the Gene ST array. Only those probe sets for genes in common across both array types were used in the analysis. This produced a set of 16,482 transcripts, and these probe sets in general exhibited a higher signal than the other probe sets on each of the arrays. 

After obtaining transcripts in common across the Exon and Gene ST arrays, we first compared smoking-induced gene expression changes in nasal epithelial cells between these two array systems. In order to identify genes that were differentially expressed between the 5 smokers and 5 nonsmokers, first, a simple Student's *t*-test was applied for data from each array platform, respectively. Then, fold changes between the log2 mean values for the smoker and nonsmoker replicates were calculated independently for each array platform. Using the matched 16,482 transcripts, we then characterized the sample correlation between these two platforms for each matched sample. 

Finally, in order to estimate the impact of different chip description files (CDFs) on gene expression measurements in our nasal clinical samples, we modified the Exon array CDF by removing probes not found in Gene ST arrays. As a result, 19,802 transcripts were left for comparison of smoking-induced gene expression changes by examining the fold changes between smokers and nonsmokers in nasal epithelium. 

### 2.5. Additional Information

All statistical analyses described previously were performed with R 2.11.0 (available at http://r-project.org/) and Bioconductor [11]. All microarray data from this study has been deposited in Gene Expression Omnibus (GEO).

## 3. Results and Discussion

### 3.1. Distribution of the Probe-Level Raw Signal on Exon and Gene ST Arrays

**Figure 1:**
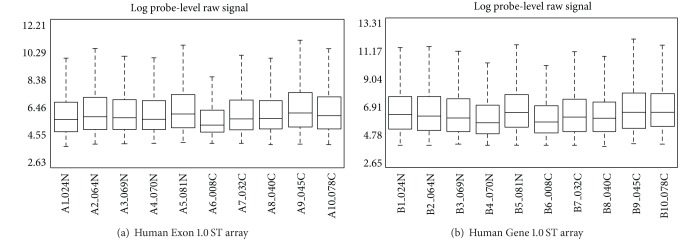
Distribution (boxplot) of the probe-level raw signal derived from (a) the Human Exon 1.0 ST array and (b) the Human Gene 1.0 ST. The raw signal distribution of each sample is similar across two platforms; for example, sample no. 6 has the lowest signal intensity distribution.

Each RNA sample collected from nasal epithelium was hybridized to both the Exon and Gene ST arrays. The distribution of the raw signal for each sample is shown in Figure 1(a) (the Exon array) and Figure 1(b) (the Gene array), respectively. Although there is a time difference for the processing and labeling between the two array systems, overall, the raw intensity for these 10 samples (5 current and 5 never smokers) is similar across the two platforms; for example, sample no. 6 has the lowest signal intensity measured by both arrays compared to the other 9 samples.

### 3.2. Sample Correlation within and between Exon Arrays and Gene ST Arrays

**Figure 2:**
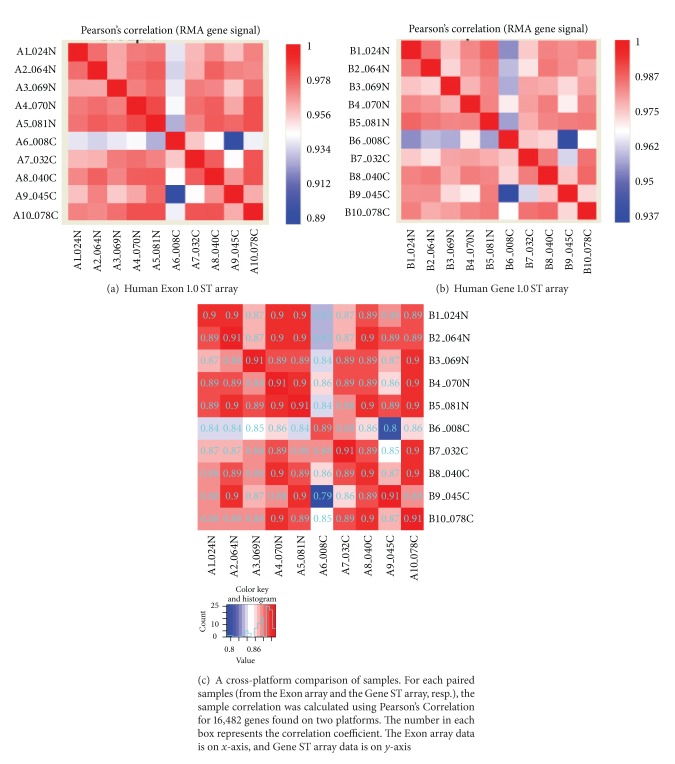
Sample correlation using RMA-normalized gene-level signals. (a) Within the Human Exon 1.0 ST array; (b) within the Human Gene 1.0 ST;(c) cross-platform sample correlation of the Exon and Gene ST arrays for 16,482 matched genes.

We first calculated the sample correlation within each platform using normalized gene level signal for all core transcripts on the Exon arrays and all transcripts on the Gene arrays. As can be seen in Figure 2, the correlation is relatively high (*r* > 0.95) for all samples with the exception of sample no.6 which is slightly less correlated with the other samples (*r* = 0.9). This could be due to its relatively low normalized signal compared to the other samples. Furthermore, the correlation is relatively higher in the Gene arrays than in the Exon arrays, perhaps due to more noisy probes on the Exon arrays (Figures 2(a) and 2(b)).

Then, in order to directly compare the correlation between the same samples hybridized on both arrays, we first identified 16,482 genes found on both platforms. The matched sample correlation between the Exon arrays and Gene ST arrays is high (≥0.9 except for sample no. 6) (Figure 2(c)). These results suggest that, compared to the Exon array, the Gene ST array is a comparable platform that is a much more cost-effective choice for well-annotated gene-level analysis. 

### 3.3. Gene-Level Differential Expression Changes on Exon and Gene ST Arrays

**Figure 3:**
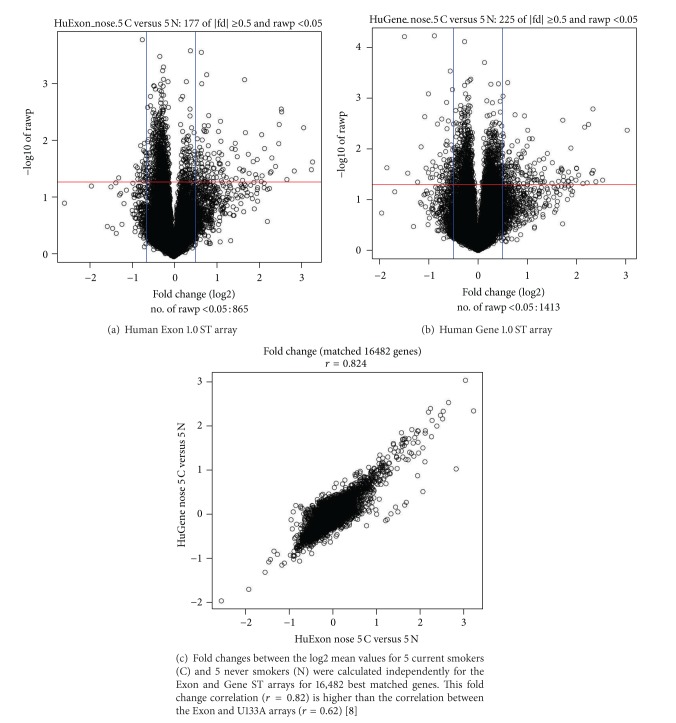
Smoking-induced differentially expressed genes using a Student's *t*-test (a) within the Human Exon 1.0 ST array and (b) within the Human Gene 1.0 ST. (c) Correlation between smoking-induced gene-expression differences (fold change) of the Exon and Gene ST arrays for 16,482 best matched gene-level probe sets.

For both Exon and Gene ST arrays, “gene-level” analysis of multiple probes on different exons is summarized into an expression value representing all transcripts from the same gene. This approach allowed us to compare genes differentially expressed between the same 5 current and 5 never smokers assayed on both arrays using a simple Student's *t*-test. Very few differentially expressed genes (DEGs) pass multiple testing (FDR < 0.05), which might be due to the small sample size in this study. However, at *P* < 0.05, many more genes are discovered from the Gene arrays (1,413 genes) than from the Exon arrays (865 genes), as shown in Figure 3(a) (the Exon array) and Figure 3(b) (the Gene array). At a significance level of 0.05, 894 (17,881∗0.05) genes are expected to be detected on the Exon array and 987 genes (19,734∗0.05) on the Gene ST array. We can see that the number of DEGs is more than expected by chance on the Gene ST array, while the number of DEGs is less than expected by chance on the Exon array using the same samples as the Gene ST array. This suggests that the Gene ST array has higher signal-to-noise ratio for gene-level analysis compared to the Exon array. This might be due to the design of the Gene ST array, on which 90% of the gene-level probe sets contain only probes that match uniquely to the genome [12], indicating less noisy probe design/contents in the Gene ST array.

After comparing statistical results of differentially expressed genes on the Exon and Gene ST arrays, we then estimated the similarities and differences in the magnitude of the gene-level fold changes between smoking status across both array platforms (Figure 3(c)). In this scatter plot, each point corresponds to a pair of transcripts for which a successful cross-chip mapping could be found (*n* = 16,482). The majority of detected gene-expression differences correlate tightly between the Exon and Gene ST arrays (*r* = 0.82; *P* < 4 × 10^−18^), which is significantly higher than the fold change correlation observed between the Exon array and the U133A array in bronchial smoking gene expression data (*r* = 0.62) [8]. This is not surprising since U133A array is one of the traditional 3' arrays, which may lack probe sets to measure the expression of some particular transcripts. However, the Gene ST arrays, whenever possible, use a subset of the same probes on the Exon array and are similar in other ways, like having probes targeting the whole transcripts (WTs), compatible with WT Sense Target Labeling and Control Reagent Kits for maximum coverage of the entire gene.

**Figure 4:**
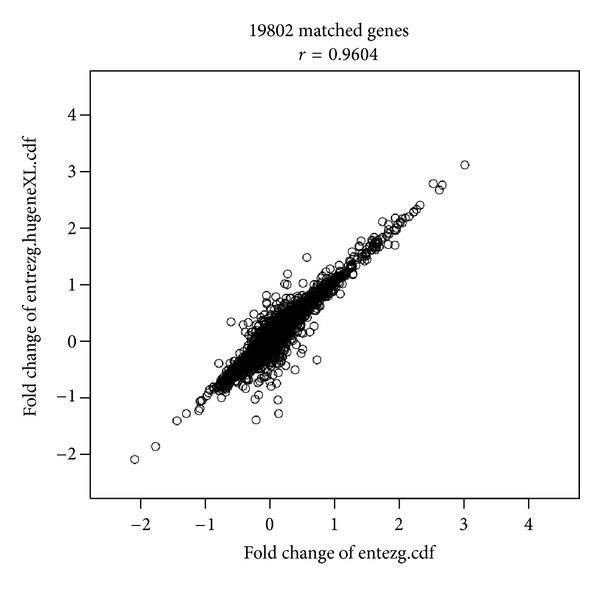
The fold change comparison of smoking-induced gene expression changes for a total of 19,802 matched genes between two different CDFs.

Finally, we applied a modified CDF to preprocess the data including background correction, normalization, and summarization, to see whether there is a difference of the impact on smoking-induced gene expression changes between utilizing different CDFs for preprocessing. As shown in Figure 4, the gene-level fold change correlation between Exon arrays and Gene ST arrays is higher with a modified CDF using a total of 19,802 matched genes (*r* = 0.96). Furthermore, 68% of DEGs defined at *P* < 0.05 overlapped, which is much higher than expected by chance.

## 4. Conclusion

In this study, we compared the performance of Exon and Gene ST arrays in our nasal epithelial samples at gene-level since the Gene ST array is designed as a focused gene level expression microarray and covers only well-characterized full-length genes by using sparser probe coverage than the Exon array. Both are WT-based arrays with a comprehensive coverage of the entire gene locus, so the Gene ST array therefore provides similar power to the Exon array for gene-level analysis in a more affordable format. But without the discovery content and full exon-level coverage, the Gene array is not designed for the high-resolution study of known and predicted alternative splicing. However, there is at least 1 probe per known exon on Gene ST arrays, so it could also be used to detect alternative splicing (AS) events. In a recent study [9], Ha et al. have shown a comparable performance of the Gene ST arrays for detecting transcript isoforms expressed differently between brain and other tissues, when compared to Exon arrays even though there are 4 probes targeting each exon on the Exon arrays.

On the other hand, the Exon array includes substantially more discovery contents, such as predicted exons and transcripts. RNA-Seq is an alternate approach for this type of discovery, but RNA-Seq has its own limitations which prevent it from having as wide an application as expression arrays, for example, much higher cost and computational and storage challenges. As one possible way to address this need, Affymetrix recently released the Whole Transcriptome Arrays, with probes targeting exon-exon junctions, noncoding RNAs, and so forth. Additional studies are needed to compare Whole Transcriptome Arrays and Gene ST arrays for well-annotated gene-level applications. 

In summary, we compared the performance of the Human Gene 1.0 ST array with the Exon 1.0 ST array for detecting smoking-related gene expression changes in nasal epithelium. The Gene ST array appears to be a reproducible platform capable of working with smaller amounts (100–300 ng) of RNAs. In a gene-level fold change comparison, we found a strong correlation between the two platforms for smoking-related gene expression changes even though the Gene ST array contains much fewer probes. These findings suggest that the Gene ST array can serve as a clinically relevant and more flexible tool (in terms of cost and input RNAs) for exploring host response to tobacco smoking in large-scale population-based studies.
